# MASTR-MS: a web-based collaborative laboratory information management system (LIMS) for metabolomics

**DOI:** 10.1007/s11306-016-1142-2

**Published:** 2016-12-27

**Authors:** Adam Hunter, Saravanan Dayalan, David De Souza, Brad Power, Rodney Lorrimar, Tamas Szabo, Thu Nguyen, Sean O’Callaghan, Jeremy Hack, James Pyke, Amsha Nahid, Roberto Barrero, Ute Roessner, Vladimir Likic, Dedreia Tull, Antony Bacic, Malcolm McConville, Matthew Bellgard

**Affiliations:** 1 Australian Bioinformatics Facility, Centre for Comparative Genomics, Murdoch University, Murdoch, WA 6150 Australia; 2 Metabolomics Australia, The University of Melbourne, Melbourne, VIC 3010 Australia; 3 Bio21 Molecular Science and Biotechnology Institute, The University of Melbourne, Melbourne, VIC 3010 Australia; 4 Metabolomics Australia, The Australian Wine Research Institute, Adelaide, SA 5064 Australia; 5 School of Biosciences, The University of Melbourne, Melbourne, VIC 3010 Australia; 6 ARC Centre of Excellence in Plant Cell Walls, School of Biosciences, The University of Melbourne, Melbourne, VIC 3010 Australia

**Keywords:** MASTR-MS, Metabolomics, LIMS, Omics

## Abstract

**Background:**

An increasing number of research laboratories and core analytical facilities around the world are developing high throughput metabolomic analytical and data processing pipelines that are capable of handling hundreds to thousands of individual samples per year, often over multiple projects, collaborations and sample types. At present, there are no Laboratory Information Management Systems (LIMS) that are specifically tailored for metabolomics laboratories that are capable of tracking samples and associated metadata from the beginning to the end of an experiment, including data processing and archiving, and which are also suitable for use in large institutional core facilities or multi-laboratory consortia as well as single laboratory environments.

**Results:**

Here we present MASTR-MS, a downloadable and installable LIMS solution that can be deployed either within a single laboratory or used to link workflows across a multisite network. It comprises a Node Management System that can be used to link and manage projects across one or multiple collaborating laboratories; a User Management System which defines different user groups and privileges of users; a Quote Management System where client quotes are managed; a Project Management System in which metadata is stored and all aspects of project management, including experimental setup, sample tracking and instrument analysis, are defined, and a Data Management System that allows the automatic capture and storage of raw and processed data from the analytical instruments to the LIMS.

**Conclusion:**

MASTR-MS is a comprehensive LIMS solution specifically designed for metabolomics. It captures the entire lifecycle of a sample starting from project and experiment design to sample analysis, data capture and storage. It acts as an electronic notebook, facilitating project management within a single laboratory or a multi-node collaborative environment. This software is being developed in close consultation with members of the metabolomics research community. It is freely available under the GNU GPL v3 licence and can be accessed from, https://muccg.github.io/mastr-ms/.

**Electronic supplementary material:**

The online version of this article (doi:10.1007/s11306-016-1142-2) contains supplementary material, which is available to authorized users.

## Introduction

Metabolomic approaches aim to detect and quantitate levels of all small molecules in a biological system and, together with other ‘omic’ approaches, can be used to generate a systems-wide understanding of biological processes. Metabolomic approaches typically involve the use of advanced mass spectrometry and NMR platforms to maximize coverage of the chemically diverse metabolites that make up biological systems. In many cases, these analytical platforms are located in institutional and/or national core facilities that offer a range of metabolomics capabilities to researchers (http://www.metabolomics.net.au, http://www.metabolomicscentre.ca, http://commonfund.nih.gov/metabolomics/index, http://www.metabohub.fr/en/, http://ec.europa.eu/research/participants/data/ref/h2020/grants_manual/hi/oa_pilot/h2020-hi-oa-data-mgt_en.pdf). These core facilities, as well as individual research groups with sophisticated metabolomics infrastructure and capability are faced with the challenge of tracking large numbers of samples and the associated metadata, and linking this information with the raw datasets generated by multiple analytical platforms, as well as processed down-stream data sets. Data handling extends beyond collection and curation of raw data, to the management of metadata that defines how the raw data is generated. Major funding agencies, such as Europe’s Horizon 2020 (http://ec.europa.eu/research/participants/data/ref/h2020/grants_manual/hi/oa_pilot/h2020-hi-oa-data-mgt_en.pdf), the NIH (http://grants.nih.gov/grants/policy/data_sharing/data_sharing_guidance.htm), The Wellcome Trust (http://www.wellcome.ac.uk/About-us/Policy/Spotlight-issues/Data-sharing/Guidance-for-researchers/index.htm) and Australia’s NHMRC (http://www.nhmrc.gov.au/guidelines/publications/r39) have established Data Management Plans that researchers are expected to follow in order to capture, store and share data generated by their grants. Scientific journals are also increasingly requesting that experimental data and metadata associated with metabolomics experiments are made available to the scientific community (http://www.nature.com/sdata/data-policies, http://www.gigasciencejournal.com/authors/instructions/research), leading to the establishment of data repositories, such as MetaboLights (Haug et al. 2012) and Metabolomics Workbench (http://www.metabolomicsworkbench.org/).

LIMS are software solutions that aim to manage the entire workflow of a laboratory. A number of LIMS have been developed or adapted from other applications for curating metabolomics experiments and data management (i.e. SetupX, Sesame). While these LIMS have features that allow capture of project metadata, experiments and samples, data storage, and data sharing they exhibit a number of limitations around their capacity to accommodate different vendor instruments and have restricted functionalities to facilitate a collaborative configuration between geographically distributed laboratories. In this paper we present MASTR-MS, the first wholly functional, open-source LIMS solutions specifically designed for metabolomics laboratories.

## Materials and methods

MASTR-MS runs as a Python (http://www.python.org) web application built on the Django (http://www.djangoproject.com) framework, utilising a PostgreSQL (http://www.postgresql.org) or MySQL (http://www.mysql.com) relational database. MASTR-MS leverages the functionality of the Django framework for user management, users permissions and security. Django is a mature web framework and provides multiple security tools and mechanisms. For example specific protection is provided against cross site scripting (XSS), cross site request forgery (CSRF), SQL injection and clickjacking. A security middleware is also used to enforce SSL/HTTPS for all traffic. MASTR-MS is built using open source components and communicates using open standards. The client side browser interface leverages Javascript and AJAX for fluid data display and submission, giving a user experience much like a desktop application, but with the flexibility of being available from any Internet connected location on any operating system, with no client side download or installation.

The DataSync Client is a small desktop application that runs on an instrument’s acquisition computer. This software constantly communicates with the MASTR-MS server and is responsible for transferring raw data from the acquisition computer to the MASTR-MS repository (Supplemental Fig. S9A). The DataSync Client is written in the Python programming language using the wxWidgets (https://www.wxwidgets.org/) GUI library and runs on Windows and Linux systems. Data is uploaded using the rsync protocol (https://rsync.samba.org/) and the libraries and plugins required for this are included in the installation package.

As the MASTR-MS server side component is written in the Python 2.7 programming language, any operating system that has Python 2.7 available for running web applications with a web server can run the application. In practice the application has only been tested on the Linux operating system and the Apache web server. For installation, operating system packages are available in RPM format for CentOS 6.5. Similarly, as the DataSync Client is also written in Python 2.7 it can run on any operating system that has Python 2.7 available. However it is typically installed on a Windows platform with a connected analytical instrument. For this reason the DataSync Client is distributed as a Windows executable (.exe) installer. The DataSync Client application is also self updating by means of a user option to upgrade to newer version if available.

## Results

MASTR-MS is a web-based LIMS solution for metabolomics laboratories. The different modules of MASTR-MS allow users to: (a) track all metabolomics samples and associated meta- analytical- and processed data sets. This starts from the capture of client/collaborator communication, the establishment of new projects, experimental design and sample definitions and the automatic capture of raw data generated by the instruments, (b) develop an electronic notebook, where users record all relevant information about projects and experiments in MASTR-MS, thus allowing multiple users to work on the same project, (c) methodically manage the vast amount of data generated by the analytical instruments, by associating it with the project, experiment and sample details and (d) facilitate collaboration between geographically distributed laboratories through the sharing of projects and experiment data. MASTR-MS is equally suited for use in either a large core facility or single-/multi-laboratory environment. Thus, both large national facilities and small individual laboratories would equally benefit from using MASTR-MS.

**Fig. 1:**
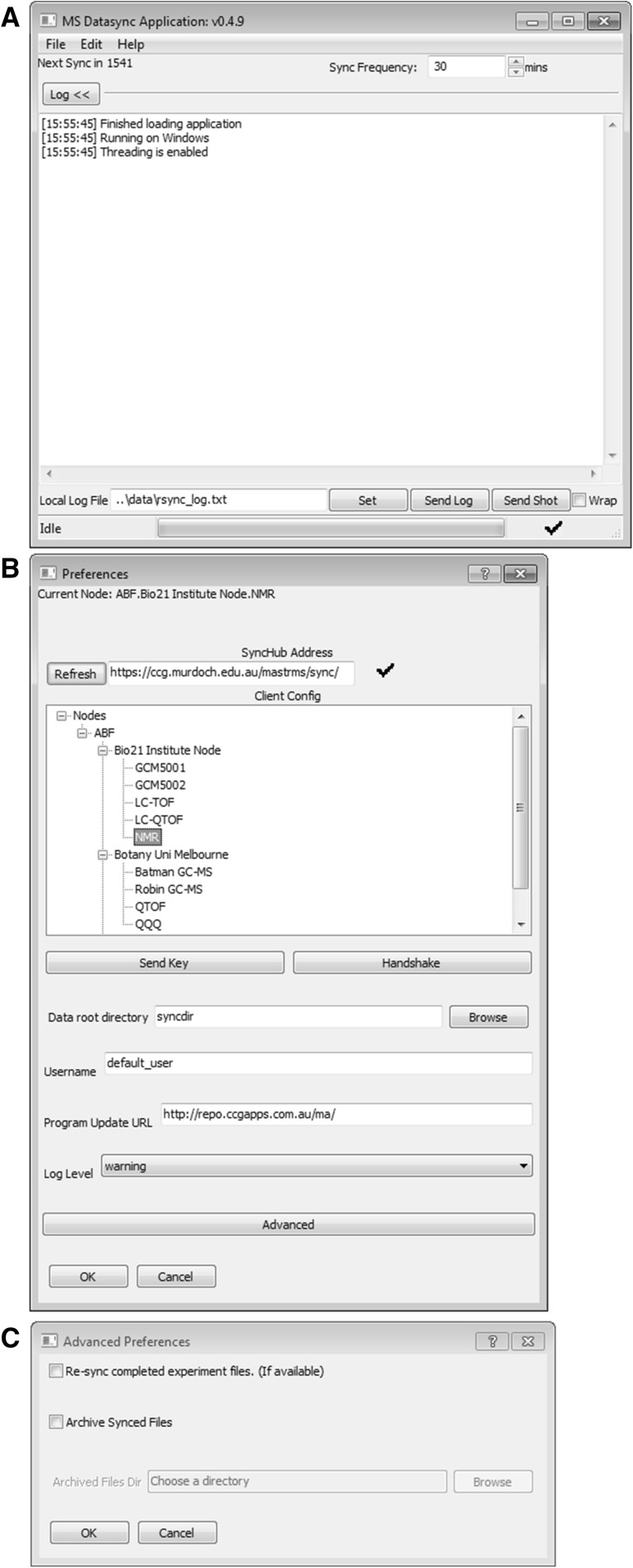
Overview of MASTR-MS system workflow

MASTR-MS comprises five major modules, (1) the Node Management System, (2) the User Management System, (3) the Quote Management System, (4) the Project Management System and (5) the Data Management System. Figure 1 shows the workflow of MASTR-MS using the different functionalities and features. These functions are described in detail below. The user is initially connected to the *Dashboard* when they first log into MASTR-MS and the functions available are tailored to the level of access of the user. The dashboard gives an “at-a-glance” summary of recent activity on the site and items requiring attention. Depending on the user’s status/level of access, the Dashboard shows Pending User Requests, Quotes Requiring Attention, Recently created / modified projects, and recently created / modified experiments.

### Node management system

This module allows the addition of multiple laboratories to be part of a single MASTR-MS network. For example, a group of geographically dispersed laboratories can have a single deployment of MASTR-MS and share projects and experiments. Such a setup would be established by the module through the generation of different nodes. On the other hand, MASTR-MS can be used within a single laboratory environment in which this module would comprise a single node.

### User management system

This module defines the different user groups used in MASTR-MS. Each user group has different privileges and permissions to access the different functionalities of MASTR-MS. In addition, this module allows the generation and management of users of the system. MASTR-MS has the following user groups:

#### Systems administrator

This user group has access to all functionalities of MASTR-MS. There would normally be one assigned Systems Administrator who would act as the query point for all other users accessing the system, although it is possible to have more than one Systems Administrator. The *Systems Administrator* has a Laboratory Name assigned to their account (like all other users), allowing a nominated user, usually a member of the organization/laboratory that is hosting the project to act as the *Systems Administrator*. The *Systems Administrator* can add new users to the system, assign user groups to any users in any laboratory, edit details of users and delete users of any laboratory.

#### Administrator

This user group has full access to all projects, experiments and experimental data, user accounts and quotes within MASTR-MS, regardless of node. This user group allows selected users to view all projects and experiments across different nodes, allowing seamless sharing and collaboration of data across nodes. Where multiple laboratories have a single MASTR-MS deployment, but prefer not to share projects and experiments, no users would be assigned the *Administrator* role.

#### Node representative

This user group has full access to quotes for their node and are the preferred contact for quotes and projects run by this node (detailed more under Quote Management System). In a multi-node setup there would typically be at least one user assigned to this group per node.

#### Project leader

This user group is able to create new projects and experiments for their node. In addition this group are able to assign staff to specific projects and experiments.

#### Staff

Users of this group are able to participate in the projects and experiments for their node.

#### Client

All other users of the system are clients. This group has no privileges other than viewing the progress of projects to which they have been assigned.

Any user of the system can update their own user record and change their password at any time.

### Quote management system

This module was designed specifically for core facilities that provide metabolomic services to client researchers. Potential clients can request a pricing quote for running samples of an experiment through the quote request system without having to sign up for an account. At a nominated stage, clients are required to register into MASTR-MS by completing a short information dialog box. This module allows collection of contact details and information about the nature of the request. Files in various formats can be attached to this module. In a multi-node facility, the user can either direct their quote to a specific node with relevant expertise or they can select “Don’t Know” to have all the Node Representatives alerted.

Quote requests made by clients and collaborators that are made through the system are tracked and marked if they have not been attended to yet, so that Node Representatives can quickly see new quotes which require attention. Quotes can only be seen by members of the node to which they were sent, unless the “Don’t Know” option was selected. Node Representatives are able to forward quotes to other nodes if required. The Node Representatives can then begin a dialogue with the potential client and with their team, clarifying the task, and providing formal quotes, attached as PDFs if necessary. Each step of the communications process is time-stamped and tracked within this module. The quote requests and any resulting quotes would eventually be associated with a project and experiment through a selection option in the Experimental Design stage. All documentation relating to the project, including the client, quote issued for the project along with the project and experimental setup is thus kept together.

### Project management system

This module allows the management of projects, experiments, sample and the creation of analytical sample runs. As detailed above, users of different user groups are able to create projects and experiments. When a project is created by either a MASTR-MS Administrator or Project Leader, it can be linked to a specific client from the user list. This allows the client to monitor how the project is progressing. Assigning a Project Manager to the project allows those users to manage all aspects of a project, experiment creation and further access control on an experiment-by-experiment basis (Supplemental Fig. S4). As sample metadata is linked to all experiments within MASTR-MS, sample classes and/or individual samples can be organised into groups and subsequently analysed on an instrument.

#### Experiment details

The Experiment Status defaults to ‘New’ when first opened and all experiment metadata is captured in this field (Supplemental Fig. S5A). Once the experiment design has been completed, the Project Manager can change the setting to ‘Designed’ to prevent further changes. The experiment can also be linked to a Formal Quote that has been previously entered in the quotes system, and if needed, can be assigned an internal job number.

#### Access control/roles

Users can be assigned to an experiment, giving them access to edit the experimental workflow and create samples and runs. Client users can also be added here giving them access to project progress information (Supplemental Fig. S5B).

#### Sample metadata

MASTR-MS uses sample metadata in order to generate sample classes, which can then be populated with individual samples (Supplemental Fig. S5C).

#### Origin/organs/parts metadata

The first metadata category is the Origin field, which contains information on sample origin and preparation (Supplemental Fig. S5D). Different metadata fields are available depending on whether the source is Microbial, Plant, Animal, Human, Synthetic, or Other.

#### Timeline/treatment metadata

MASTR-MS also accepts time course and treatment metadata, where samples have been collected over multiple time points, or after different experimental treatments. The Origin, Timeline, and Treatment fields are then used to automatically generate sample classes.

#### Sample preparation

MASTR-MS allows an upload of a Standard Operating Procedure (SOP) document to be associated with an experiment. Multiple SOPs can be uploaded, and additional notes recorded for each. A SOP is linked with methods used during runs at the time of setting up a run. The SOP is linked at the experiment level and the option of choosing methods is provided under the runs level. This is to incorporate the option where a user would like to run multiple methods during a run (either by resampling the same vial or from a different vial).

#### Automatic sample class generation

Based on the metadata entered in the Origin, Timeline, and Treatment steps, sample classes are automatically generated based on permutations of the available metadata (Supplemental Fig. S7A). If abbreviations have been provided for a particular metadata category, these will be used during sample class generation. Samples can then be created in each sample class.

Samples can then be viewed and collected together to form a run on a designated analytical instrument platform (Supplemental Fig. S7B). Additional sample information can be imported via CSV and exported from MASTR-MS in the same way. Samples can be randomised before putting them into a run if desired.

#### Runs

Selected samples are added to a new or existing run by clicking the ‘Add Selected Samples to Run’ button. This will display a dialog allowing the user to add either the samples to a new run or to any previous run which is still unlocked for editing (Supplemental Fig. S8A). Runs continue to be unlocked as long as a work-list has not yet been generated for them. Locked runs can be edited and reused if needed using the “Run Cloning” feature, which will duplicate the Run data into a new unlocked Run.

#### Work-list generation

The goal of run configuration is to streamline sample analysis and generate instrument worklists in a convenient and flexible manner. After sample data has been added to a run, the order and sequencing of additional run elements (Sweeps, Solvents, etc) can be added via the Rules Generator.

The Rules Generator provides a customisable set of steps (rules) which dictate how work-lists are built. It consists of a Start Block, Sample Block, and End Block, each of which allows the insertion of non-sample components into the work-list. These include Pooled Biological QC, Sweep, Reagent Blank, Solvent Blank and Pure Standard.

The sample block, containing the experiment samples, allows “*n”* components to be inserted every “*m”* samples, in random or position order (Supplemental Fig. S8B). Once all three blocks have been designed, the Rule Generator can be enabled, disabling further editing and making the Rule available for inclusion in Run work-list generation. Rule Generators can be restricted to use by a single user, an entire node, or everybody on the system. Enabled Rule Generators can be cloned in order to generate a new version, which can then be extended and modified.

To generate a work-list within a Run, the user selects an instrument (configured and made available by Administrators) and a Rule Generator if needed and clicks the ‘Generate Work-list’ button. Once the work-list is generated, further modification of the Run is not possible. The specific work-list format is customisable by site administrators to provide flexibility among various instrument models. Once the work-list is generated it can be used with the instrument to automate the raw data collection process.

### Data management system

This module facilitates the capture and storage of raw data produced by the instruments. The raw data is captured by the DataSync Client as detailed below and is linked to associated project and experiment details. In addition, post-processed data and any other related files such as presentations, reports and papers can be linked to the data.

#### Data acquisition and the datasync client

The DataSync Client allows data to be transferred from connected instruments at nominated frequencies and will run in the background of the acquisition computers as an icon in the System Tray. The software is fully integrated with the MASTR-MS web application. When data synchronization is requested, either scheduled or manually, the DataSync Client communicates with the MASTR-MS system to query all incomplete experiment runs which have been configured for the connected instrument. It then searches the acquired data for required files and transmits them to the MASTR-MS repository via a configurable rsync transport, allowing compression and check-summing for efficient data transfer. The configuration options for individual DataSync Nodes are fully configurable via the MASTR-MS administration interface.

To enable DataSync Client uploads on the instrument, the user simply selects the connected instrument from the list which has been configured on the MASTR-MS system and enters the Rsync username which they have been assigned (Supplemental Fig. S9B). OpenSSH Public Keys can be uploaded to the MASTR-MS system for secure password-less usage, which allows the client to run seamless automated data uploads without need for operator intervention.

The DataSync Client can also be configured with some advanced options. Data archival allows the raw sample data to be automatically replicated in a specified location (e.g. on another hard disk) once confirmation of upload has been achieved, allowing the original data to be deleted if desired.

The software can also be forced to re-synchronise experiment data that has been marked as complete in case the need arises (Supplemental Fig. S9C).

#### Run progress

As data is synced with the MASTR-MS system, run progress is updated to reflect the number of confirmed files acquired versus the number expected. Once the MASTR-MS system has confirmed that Run progress is at 100%, the Run is marked complete and the run data is available to authorized users for download. Component files and Sample files are available for download separately and Sample files can be packed into compressed archives (zip, tar.gz, tar.bzip) for efficient download, to minimise download sizes.

MASTR-MS is designed in a generic form such that it accommodates the automatic capture and transfer of any type of data from an acquisition computer to the server. This feature allows MASTR-MS to be used with instruments from different vendors with different file types.

## Discussion

The systematic tracking, analysis and sharing of complex datasets generated by high through-put omics technologies such as those used in metabolomics represents a major and expanding challenge. Reliance on outdated methods for recording information about projects, experiments, samples and instruments is cumbersome and error-prone. The methodical management of lab data can be achieved by software solutions such as LIMS and electronic notebook systems. An ideal LIMS solution should be able to manage users and user privileges of the lab, manage the setting up of projects, experiments and samples and manage the resulting data. It should be able to facilitate sharing of meta/experimental data to other collaborating laboratories. The advantages of using task specific LIMS over the old manual lab book or even simple spreadsheets are enormous. With well designed systems such as LIMS solutions, search and retrieval becomes easy and efficient, especially in a lab that has been operating for several years, thereby having collected information on hundreds of projects and experiments. In addition, security plays an important role in LIMS solutions. Access to information and data about projects, experiments and samples would be controlled to be accessed only by authorised individuals. Finally, all information can be backed up to secure locations, thereby reducing the risk of accidental loss of data (Table 1).

**Table 1:** User roles and access privileges

User type	Access privilege
Administrator	Complete Read, Write access to all modules to all Nodes
Node representative	For their specific node, complete Read, Write access to all modules
Project manager	For their specific node, Read, Write access to only project and experiments associated to them
Lab assistant	For their specific node, Read, Write access to only experiments associated to them
Client	Read access to only experiments associated to them

MASTR-MS is a comprehensive web-based LIMS solution that has been tailor-made for metabolomic experiments and is suitable for implementation within a single laboratory environment or across a multi-node research consortium/core facility. It (a) captures the entire lifecycle of a sample from project and experimental design to the automatic capture and methodical storage of raw data generated by the multiple analytical instruments, (b) stores metadata about projects, experiments and samples and links the raw data with the metadata, (c) acts as a comprehensive electronic workbook, (d) acts as a storage solution for the vast amount of high throughput data generated by metabolomic experiments and (e) facilitates collaboration between different laboratories.

### Scope of MASTR-MS

MASTR-MS efficiently manages the lifecycle of a sample, capturing information from client communication through to establishing projects, experiments, samples and continuing to automatic capture of raw data from the analytical instruments. MASTR-MS also stores processed data along with results of any statistical analysis and project reports. By design, MASTR-MS does not provide tools for data processing or statistical analysis, allowing researchers maximum flexibility for data processing and analysis, while allowing processed data to be imported and linked to a raw data. .

An important function of MASTR-MS is to act as an electronic laboratory notebook. To facilitate this, information is collected through free-flowing text fields. The advantage of this approach is that it allows the users to enter the same types of information that they would enter in their traditional lab notebook. The limitation behind this approach is that the entries are not controlled for ontologies and therefore adopting to standards becomes challenging. Changing the free text entry to controlled vocabulary, incorporating the current MSI standards as well as adopting the Metabolomics community standards (ISA-Tab, mw-Tab) will be considered in future iterations of MASTR-MS.

### Comparison to similar softwares

MASTR-MS offers a number of features that distinguish it from other metabolomics LIMS systems such as SetupX and Sesame. SetupX (Scholz and Fiehn 2007) is a web-based metabolomics LIMS solution that is XML compatible and built around a relational database management core. It is particularly oriented towards the capture and display of GC–MS metabolomic data through its metabolic annotation database, BinBase (Skogerson et al. 2011). SetupX is able to handle a wide variety of BioSources (spatial, historical, environmental and genotypic descriptions of biological objects undergoing metabolomic investigations) and Treatments (experimental alterations that influence the metabolic states of BioSources). Compared to SetupX, MASTR-MS has not associated its input fields to ontologies, although it is intended that this will be incorporated into future versions of MASTR-MS as international standards are increasingly being adopted. Compared to SetupX, MASTR-MS offers the following advantages. It is able to cover multiple collaborating labs with a single deployment; lab-based users can generate the sequence list of samples to be run in the analytical instruments, thereby saving time and reducing the possibility of human errors; raw data generated by analyses is automatically captured by MASTR-MS; the extensive user management system and the ability of collaborators and clients to interact with the nodes using the Quote Management System.

Sesame (http://www.coreinformatics.com/) is also a web-based, platform-independent LIMS. It is based on Java CORBA, a commercial and open source RDBMS-es, and was originally developed to facilitate NMR-based structural genomics studies (http://grants.nih.gov/grants/policy/data_sharing/data_sharing_guidance.htm). The Sesame module for metabolomics is called ‘Lamp’. The Lamp module was originally designed to process NMR metabolomic analyses of *Arabidopsis*, although it is flexible enough to be easily adapted to other biological systems and other analytical methods. It consists of a number of different ‘Views’ which provide details about the data, the instruments, and system resources used in a given study. In Sesame, the Views are designed to operate on various kinds of data, and facilitate data capture, editing, processing, analysis, retrieval and report generation. Sesame is a broad LIMS solution whose origins are in structural and functional proteomics, managing data from NMR platforms. Lamp, the module of Sesame that manages metabolomics data is one of nine application modules of Sesame and was originally designed to manage information about the expression and purification of proteins and store this information. As Sesame and Lamp were not originally designed for metabolomics, its functions and features do not directly reflect the workflow of a typical metabolomics experiment. For example, even though Sesame has an extensive user management system, it does not have the functionalities of MASTR-MS that was specifically designed for metabolomics such as, an exhaustive project, experiment and sample management system, the ability of users of the lab to generate the sequence list of samples to be run in the analytical instruments, automatic capture of raw data from instruments and the ability of collaborators and clients to interact with the nodes using the Quote Management System.

In addition to the above discussed open source solutions, there are several commercial LIMS solutions such as MetaboLIMS from Core Informatics (http://www.coreinformatics.com/), MetLIMS from BioCrates (http://www.biocrates.com/) and Clarity LIMS from GenoLogics (https://www.genologics.com/editions/clarity-lims-gold/). Due to their commercial nature, their functions and features are not readily available to compare against MASTR-MS.

## Conclusion

This paper describes MASTR-MS, a new, fully integrated, open-source LIMS solution specifically designed for metabolomics laboratories. MASTR-MS can be used to track and share metabolomics experiments within a single laboratory or across large collaborative networks. Its comprehensive functions and features enable researchers and facilities to effectively manage a wide range of different project and experimental data types and facilitate the mining of new and existing datasets. The generic design of the data management component of MASTR-MS ensures that it can be used with instruments from different vendors. In addition, we have found that MASTR-MS can provide a LIMS solution for other data-rich technology platforms, such as proteomics, NMR and imaging facilities. MASTR-MS already has considerable community support and new features will continuously be incorporated, including the capacity for researchers to directly upload their metadata and data to public metabolomics repositories such as MetaboLights and the Metabolomics Workbench. In addition, a reporting and export function is being developed at the user level, enabling the user to query the system and download data. In order to make automatic querying and retrieval easy, an API for MASTR-MS is being planned as well.

## Availability and requirements

- Project name: MASTR-MS
- Project home page: https://muccg.github.io/mastr-ms/.
- Operating system(s): Server Installation: Centos 6.x (x86_64); Client: Any operating system and modern web browser can be used as the web client to access MASTR-MS; DataSync Client: Linux or Windows
- Programming language: Python 2.7
- Software requirements: Apache 2.2 or higher, PostgreSQL 8.4 or higher
- License: GNU GPL v3
- Any restrictions to use by non-academics: See GNU GPL v3

## Electronic supplementary material

Below is the link to the electronic supplementary material.

Supplementary material 1 (JPG 137 KB)

Supplementary material 2 (PNG 48 KB)

Supplementary material 3 (PNG 6836 KB)

Supplementary material 4 (PNG 3593 KB)

Supplementary material 5 (PNG 2913 KB)
