# Supplementary figures and images for: MASTR-MS: a web-based collaborative laboratory information management system (LIMS) for metabolomics
Source: Metabolomics. 2016 Dec 27;13(2):14. doi: 10.1007/s11306-016-1142-2 (PMC5192047; doi:10.1007/s11306-016-1142-2)

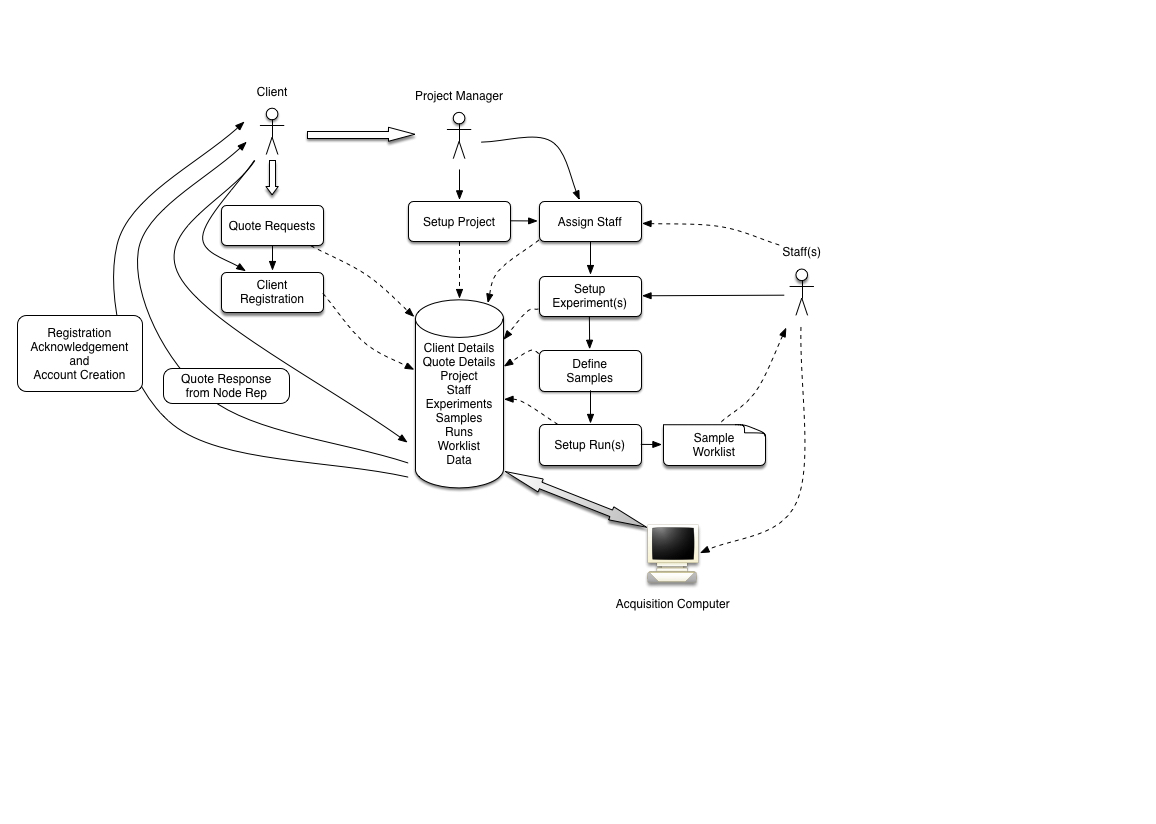

Supplement: Supplementary file 1 — Supplementary material 1 (JPG 137 KB) [file 11306_2016_1142_MOESM1_ESM.jpg]

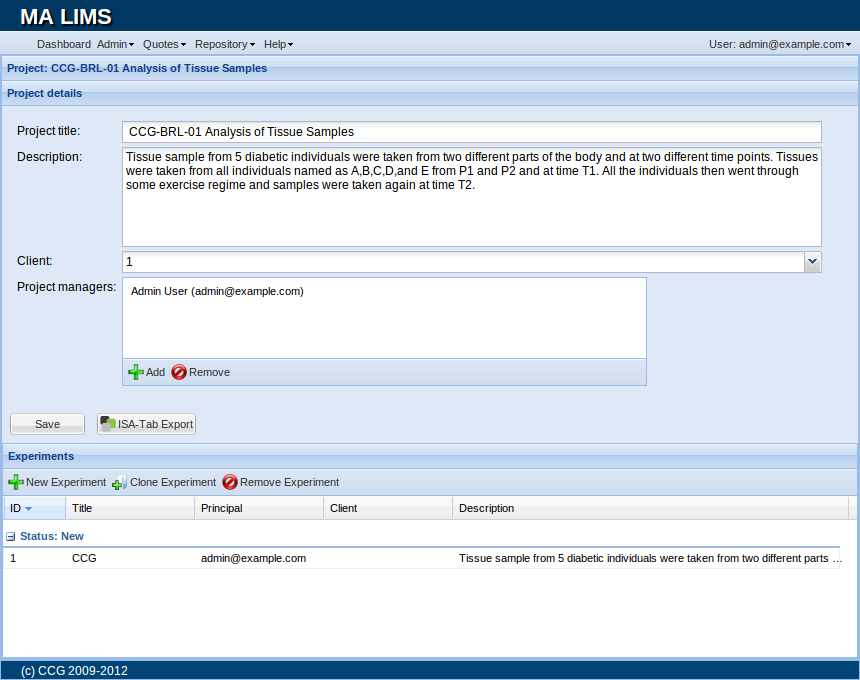

Supplement: Supplementary file 2 — Supplementary material 2 (PNG 48 KB) [file 11306_2016_1142_MOESM2_ESM.png]

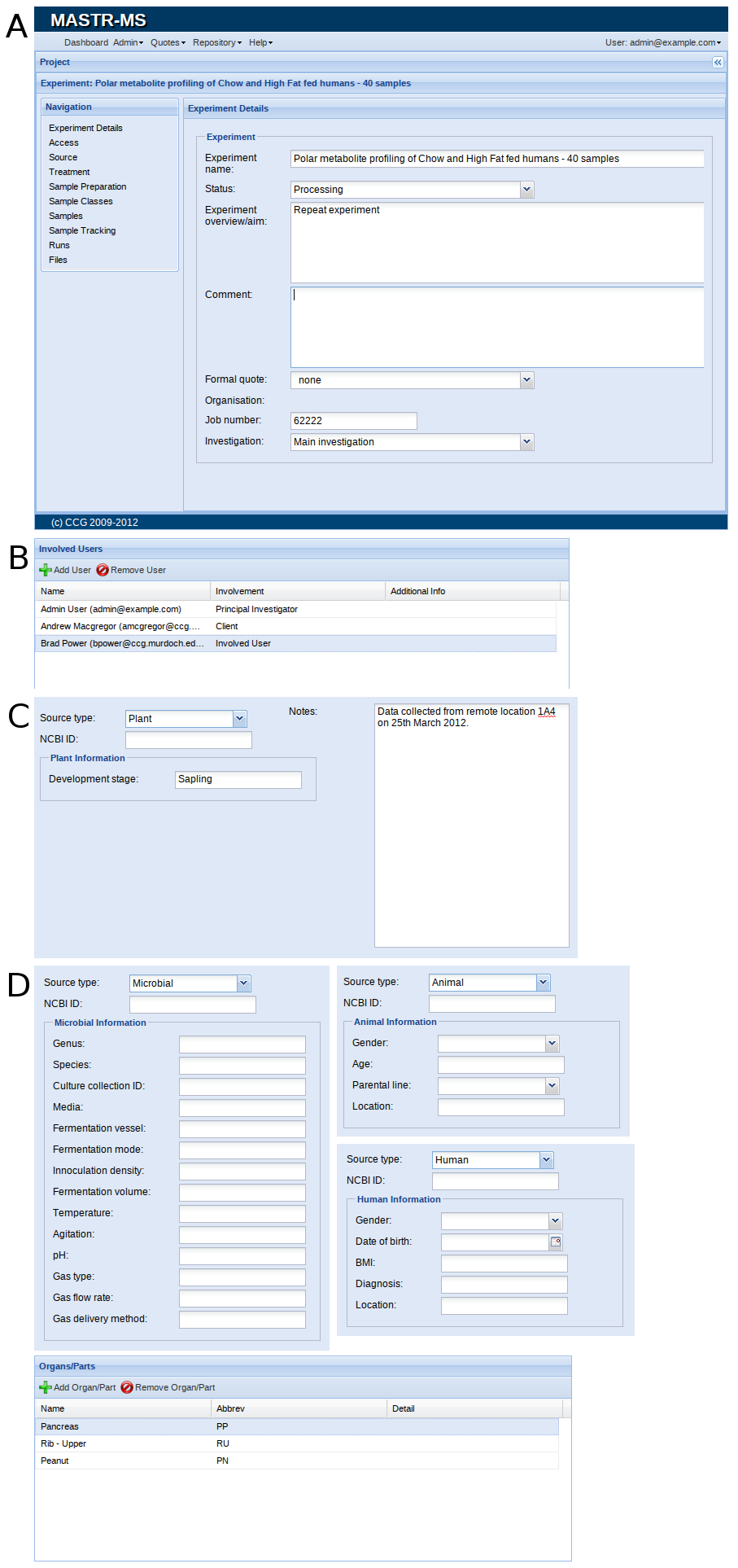

Supplement: Supplementary file 3 — Supplementary material 3 (PNG 6836 KB) [file 11306_2016_1142_MOESM3_ESM.png]

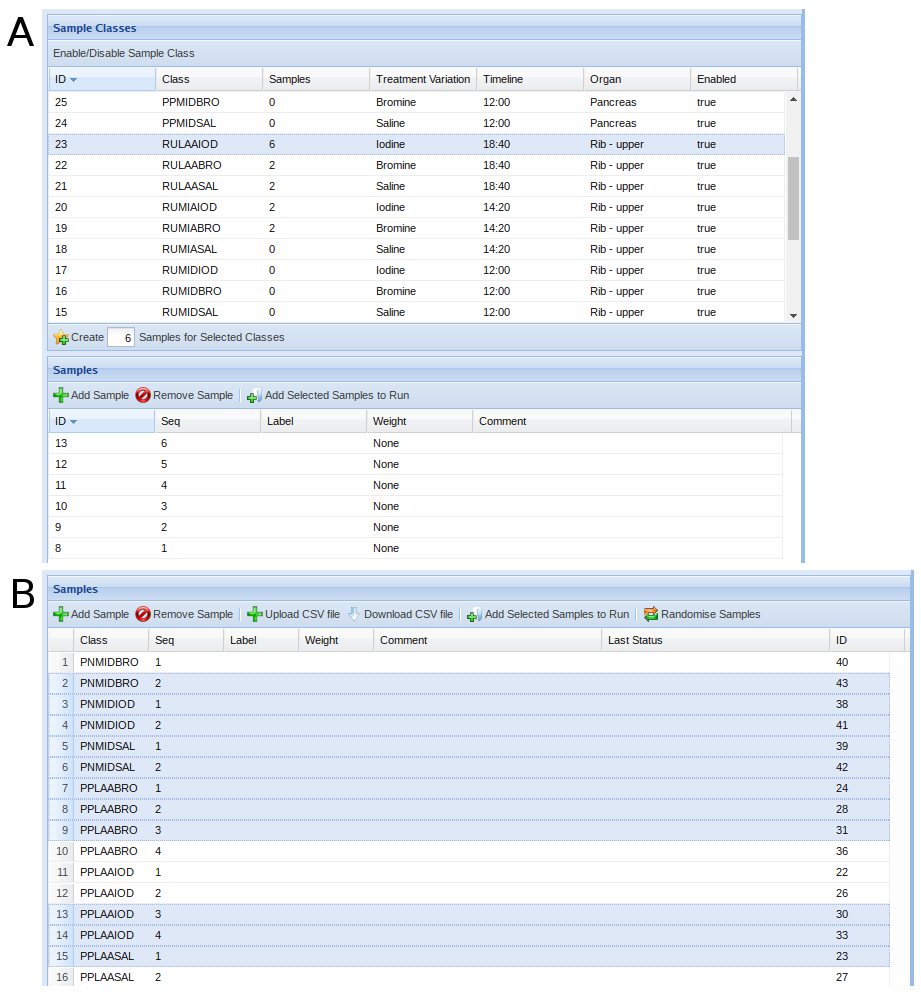

Supplement: Supplementary file 4 — Supplementary material 4 (PNG 3593 KB) [file 11306_2016_1142_MOESM4_ESM.png]

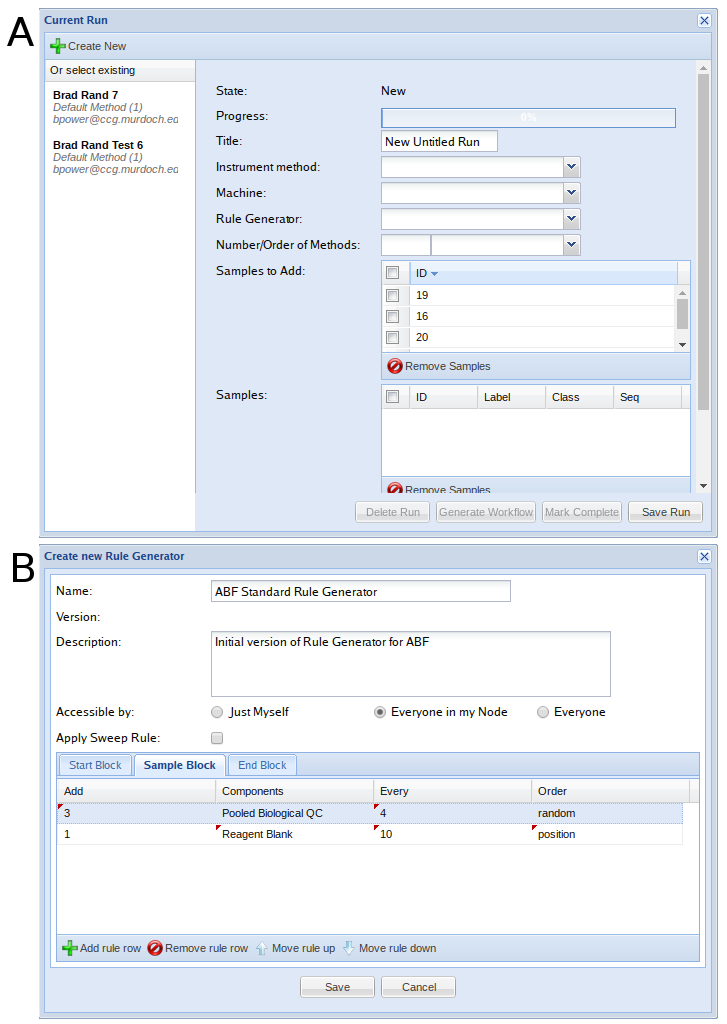

Supplement: Supplementary file 5 — Supplementary material 5 (PNG 2913 KB) [file 11306_2016_1142_MOESM5_ESM.png]
